# Who Are the Beneficiaries of China’s New Rural Pension Scheme? Sons, Daughters, or Parents?

**DOI:** 10.3390/ijerph16173159

**Published:** 2019-08-29

**Authors:** Zhaohua Zhang, Yuxi Luo, Derrick Robinson

**Affiliations:** 1 College of Economics and Management, Shandong Agricultural University, Tai’an 271018, China; 2 School of Economics and Management, Guangxi Normal University, Guilin 541004, China; 3 Division of Agriculture and Natural Resources, University of California, San Diego, CA 92123, USA

**Keywords:** New Rural Pension Scheme, regression discontinuity, family-based eldercare, intergenerational relationship, elderly health

## Abstract

By applying a fuzzy regression discontinuity design, this study investigates whether sons, daughters, or parents are the beneficiaries of China’s New Rural Pension Scheme. Using data drawn from the China Health and Retirement Longitudinal Survey, our results indicate that pension income crowds out approximately 27.9% of the monetary support from adult sons and decreases the likelihood that adult sons live with their parents by 6.5%. However, we do not find a significant effect of pension income on the likelihood that adult daughters live with their parents. In regards to the well-being of parents, which is measured by consumption and health outcomes, the results show that pension income increases food and non-food consumption by 16.3 and 15.1%, respectively, and improves the psychological health of the elderly. Accounting for the different effects of pension income for those with different income levels, our results show that the New Rural Pension Scheme only has a significant effect on the poor elderly.

## 1. Introduction

With the largest elderly population in the world, China is aging at an unprecedented level. With the majority of the elderly living in rural areas, policymakers are turning their attention toward how to ensure the well-being of the elderly in these areas. In China, especially in rural China, the family is traditionally responsible for taking care of their elders as there is no established, sustainable social security system. However, two major factors have placed pressure on this traditional family-based system: the implementation of the one-child policy during the last three decades and the rural-to-urban migration of young adults beginning in the 1990s [1,2]. In order to ensure the well-being of the aging population and establish a sustainable social pension system in rural China, the Chinese government launched the New Rural Pension Scheme (NRPS) in late 2009, and this program may have important implications for the traditional family support system.

To alleviate the pressure stemming from an aging population, social pension programs are adopted as an important policy instrument in both developed and developing countries [3]. The NRPS is one of the social pension programs launched by the Chinese government for the purpose of guaranteeing the basic needs of rural residents when they are old. With the introduction and expansion of the NRPS, there is a growing research interest in exploring whether this program can reach its expected goals [2,4,5]. With considerable attention being paid to intergenerational economic transfer and living arrangements [6,7], few studies have investigated the differences in benefits that the elderly and their adult children receive from the NRPS. Given the cultural heritage and norms surrounding “Filial Piety,” adult children are responsible for taking care of their elderly parents in China. This informal family support remains the primary source of support for the elderly, especially for the rural elderly. The introduction of the NRPS has important implications for traditional family support. Pension payments raise the income of elderly parents, which is a substitute for the monetary support from adult children, thus relieving adult children’s financial burden [5]. Pension income from the NRPS also increases elderly parents’ economic independence, which enables adult children to live separately from their parents and migrate to urban areas to pursue a better life. Therefore, the benefits received by adult children from the NRPS in this study are measured by both intergenerational transfer reduction and changes of living arrangement. The benefits for elderly parents are measured by consumption and health outcomes. The NRPS “windfall income” relaxes the budget constraints of the elderly, leading to a change in their consumption pattern. The change in consumption pattern may further affect the physical health of the elderly by allowing them to purchase and consume more nutrient-rich food. Additionally, with pension income, the elderly are less likely to participate in physically demanding work that adversely affects health. Besides physical health, we are also interested in the impact of the NRPS on the psychological health of the elderly.

In addition to exploring the differences in benefits that the elderly and their adult children receive from the NRPS, this paper also disaggregates the children’s benefits by gender. A traditionally patrilineal culture has led parents in most parts of rural China to rely on their sons rather than daughters for support, although in some developed counties sons and daughters provide similar levels of support [8]. Therefore, we assume that pension income paid to elderly parents has different impacts on sons and daughters. To test this hypothesis, this study examines the effects of the NRPS on the well-being of sons and daughters, using a rich micro survey dataset on China’s elderly population from the China Health and Retirement Longitudinal Study (CHARLS). Since pension is granted based on age, to disentangle the effect of pension receipt from other age-related factors that affect traditional family support, a regression discontinuity (RD) design is employed in this study. Using a rich survey of the elderly population in China, our results indicate that sons, who carry the bulk of responsibility for taking care of their elderly parents, are more responsive to their parents’ pension income than daughters. The NRPS crowds out approximately 27.9% of the monetary support from adult sons and decreases the likelihood that adult sons co-reside with their parents by 6.5%. However, we did not find any significant effect of pension on the likelihood of co-residency between adult daughters and their parents. Parents’ well-being analysis shows that pension income increased parents’ consumption level and improved their psychological health status. To capture the differences and relative importance of pension for the elderly of different income levels, we divided the sample into two subsamples: the poor elderly and the non-poor elderly. The results of this indicate that the NRPS only has a significant effect on the poor elderly, which is not surprising given that a small pension income will be more beneficial to those with a lower income level.

The remainder of this paper is organized as follows: Section 2 describes the materials and methods used in this study. Section 3 presents the estimation results, which is followed by the discussions section. Section 5 provides concluding remarks.

## 2. Materials and Methods

### 2.1. Pension Programs in Rural China

Unlike developed countries such as the United States and countries in Europe, where social safety net systems cover the majority of the elderly before the aging of the population [3], China is aging in relatively less-developed political and financial institutions. A pilot social pension program that targeted the rural elderly was introduced at the beginning of the 1990s under the supervision of the Ministry of Civil Affairs [9]. However, this program placed financial responsibility primarily on individual contributions, leading to a very low take-up rate. Coupled with wide concerns about the financial sustainability and effectiveness, the central government decided to terminate this program in 1999 [10]. As China is aging rapidly, it is crucial for policymakers to introduce an efficient and sustainable social pension program aimed at supporting the elderly population in rural China. In September 2009, China launched the NRPS for rural residents, beginning with 320 pilot counties and eventually expanding to almost all 2853 counties by 2012 [6].

Different from the previous pilot pension program, the NRPS pension fund consists of two parts: an individual premium and subsidies shared between central and local governments. In regards to the individual premium, beneficiaries can select a premium level from five categories: 100, 200, 300, 400, and 500 CNY per person per year. Though local governments may introduce more categories to adjust for higher or increasing costs of living, 100 CNY has proven to be the most popular premium choice among the participants [10]. The minimum subsidy amount provided by central and local governments should be no less than 30 CNY per person per year, and the maximum amount is capped at 50 CNY. The subsidies are financed by both local and central government. The percentage of premium subsidy provided by the central government varies across provinces based on economic status. Generally, this percentage can be classified into 4 categories: 20, 40, 60 and 80%. The higher the individual premium one chooses to pay, the greater the subsidy match embodied in the government premium contribution. The individual premium and the government subsidies form the NRPS pension account.

Rural residents over the age of 16 who have not participated in the basic old-age pension in cities may voluntarily participate in the NRPS. The NRPS pension-eligibility is age determined. Participants aged below 45 when the NRPS was launched are required to contribute at least 15 years to be eligible for pension benefits at the age of 60. Those aged 45–59 at the time of the NRPS rollout can contribute during their working lives or pay a lump sum to cover any shortfall in the vesting period of 15 years of contribution to be eligible for pension payments. The elderly aged 60 and over during the NRPS implementation can directly receive the basic pension benefit. The NRPS benefit includes a noncontributory (or basic) pension and an individual contributory pension account. The basic pension is fully financed by the government and is available to all participants when they are 60. The level of the basic pension varies across provinces, with a minimum payment of 55 CNY (around 9 USD) per month and a maximum payment up to 360 CNY (around 60 USD) for some wealthier provinces (e.g., Shanghai, Beijing). The minimum value of 55 CNY per month is close to the 2008 poverty threshold set at 783 CNY. This benefit payment method represents the first time that the Chinese government has undertaken major financial support for a rural pension system [11]. The accumulated contribution in the individual account will be paid back to the pensioner with 139 months starting from the age of 60. Since both central and local governments play an important role in the NRPS, this program has prompted more rural resident participation and has performed much better than the previous pilot program. Chen et al. (2019) [12] showed that total pension benefits, including both the basic pension and a possible individual account, accounts for approximately 15 percent of China’s average earned income.

### 2.2. Data and Variables

#### 2.2.1. Data Source

This study utilizes data drawn from CHARLS, which is conducted by Peking University. The CHARLS is a national survey designed to gather personal and family information about the population aged 45 and older. The baseline wave was fielded in 2011, which is followed up every other year. People aged 45 and older are randomly selected to be interviewed. The CHARLS dataset includes demographic background and family information, health status and functioning, health care and insurance, pension income and assets, and other related information. Its rich information regarding pension receipt and intergenerational relationships makes it an ideal dataset for evaluating the performance of the NRPS. In 2009, only 320 counties (accounting for 10% of the total number of counties) were selected as pilot counties of the NRPS. It was not until 2012 that the NRPS covered the whole country. To avoid policy selection bias, this study applied the data from the latest wave of the CHARLS, which was conducted in 2015, to update the literature evaluating NRPS performance. Since the NRPS targets the elderly population in rural areas, we restricted the sample to only the rural elderly, excluding observations for pension programs outside of the NRPS.

#### 2.2.2. Measurement of Benefits for Children

The benefits received by adult children from the NRPS are measured by the impact of pension income on intergenerational transfers and living arrangement. To collect information on pecuniary transfer, respondents were asked to recall the financial support they received in the previous 12 months from their adult children who lived elsewhere. In China, the monetary transfer to elderly parents is primarily from their adult children who do not live with them. Therefore, transfers from children who live with their parents are beyond the scope of this study. Evidence to date [13,14] suggests that public pension has crowed out private transfer from adult children, and the extent of this crowd out is the interest of our study. The CHARLS asked each respondent detailed questions about each of their children, regardless of whether they live together or not. Intergenerational living arrangements are measured by whether elderly parents co-reside with adult sons or with adult daughters. Due to rural China’s patriarchal tradition, adult sons play a more prominent role than daughters in providing care to elderly parents. Therefore, we hypothesize that pension income may induce different effects on adult sons and adult daughters. Accordingly, we classify monetary transfers and co-residence decisions by children’s gender.

#### 2.2.3. Measurement of Benefits for Aging Parents

Benefits for elderly parents are measured by consumption and health outcomes. Data on consumption is drawn from the questionnaire section on income, expenditures, and assets. In addition to food consumption, we investigate whether pension income increases expenditure on non-food items. Non-food items include clothing and bedding, travel, heating, durable goods, education and training, medical treatment, fitness, beauty, automobiles, property management fees, and donations.

Health information about the respondents was reported in the questionnaire of health status and functioning. Both physical and psychological health measures are used to assess the health outcomes of the NRPS. Physical health is measured by the ability to perform instrumental activities of daily living (IADL), which is a binary indicator taking on a value of 1 if a respondent reports that they can finish 6 daily activities without assistance. These 6 activities are cleaning and maintaining the house, preparing meals, shopping for groceries and necessities, using the telephone or other form of communication, managing money, and taking prescribed medications. Psychological health is measured using an index of depression, consisting of 10 questions. The 10 questions are: (1) “Are you bothered by things that don’t usually bother you?”; (2)“Do you have trouble keeping your mind on what you was doing?”; (3) “Do you often feel depressed?”; (4)“Do you feel everything you do is an effort?”; (5) “Do you feel hopeless about the future?”; (6) “Do you feel fearful”; (7) “Is your sleep restless?”; (8) “Do you feel happy?”; (9) “Do you feel lonely?”; (10) “How do you rate your life at present?”. Following the literature, we use the sum of all the responses as a measure of mental well-being; the response to each question ranges from 0 to 3, and higher scores indicate lower psychological health [7]. The individual questionnaire items are demonstrated to be identical to those used in standard psychological health measures in the literature [15,16].

Our study sample contained 9324 observations. The summary statistics of all the variables are presented in Table 1, showing that 46.1% of the NRPS participants received a pension income. On average, the food expenditure of the rural elderly accounted for approximately 46% of the total expenditure (food expenditure + non-food expenditure). Due to traditional patriarchy and gender discrimination in rural China, disentangling the effects of pension income on sons from that on daughters is critical to our analysis. Table 1 shows that the average transfer from adult sons is approximately twice that from adult daughters. Additionally, 32.3% of the respondents were living with adult sons, while only 6.5% were living with adult daughters. This indicates that, currently, parents still rely on sons for elderly care in rural China. These sons generally provide parents with two caregivers (his bride and himself). Meanwhile, daughters are expected to care for their in-laws, leaving their parents with no caregivers. Other individual characteristics included are age, gender, educational attainment, and household income. Approximately 67% of the respondents had no formal education or had only attended elementary school. Since a large proportion of the respondents had little education, interviewers provided assistance if they experienced difficulties in understanding the questionnaire.

### 2.3. Model Specification

**Figure 1:**
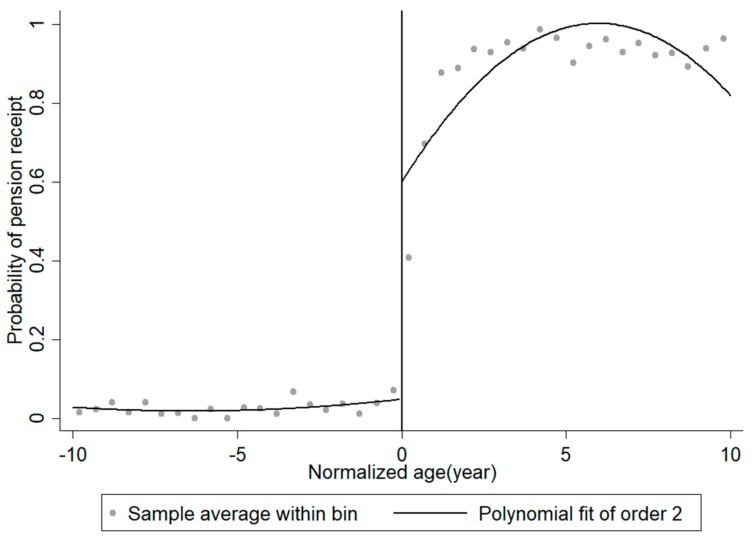
Pension receipt according to normalized age. Source: China Health and Retirement Longitudinal Survey (2015) [17]. Notes: Figure 1 describes the relationship between rate of pension receipt and respondents’ normalized age, using the sample of respondents aged between 50 and 70. The dots show the sample mean of the indicator variable for receiving a pension (1 if receiving pension, 0 otherwise) in each half year. The line is a polynomial fit of order 2.

Given the discontinuity in age in the benefit of the NRPS, this study uses a RD design to overcome the endogeneity of pension receipt. Assuming other household and individual characteristics are smoothly distributed according to age, the RD design used in this study allows us to isolate the causal link between the NRPS and the outcomes of interest. Generally, the RD design can be classified into two categories: sharp RD designs and fuzzy RD designs. The crucial assumption of a sharp RD design is that there is a discontinuity in the probability of assignment from 0 to 1. Meanwhile, a fuzzy RD design does not require a sharp discontinuity in the probability of assignment, but rather is applicable as long as the probability of assignment is different. In the context of the NRPS in China, NRPS participants will begin receiving pension payments when they reach age 60. However, due to different payment timelines in each local government (some local governments pay the pension every month, but many local governments prefer to pay at the end of each season or at the end of the year), many elderly people do not receive their pension income immediately after their 60th birthday. Therefore, some respondents reported in the survey that they did not receive their pension payment even though they were age eligible. As plotted in Figure 1 approximately 60% of the respondents who turned 60 received their pension payments, indicating that a fuzzy RD design is more appropriate for our analysis.

In the fuzzy RD design, the ratio of the jump in the regression of the outcome on age to the jump in the regression of the treatment indicator on age is described as an average causal effect of treatment [18]. Formally, the fuzzy RD estimate is:

(1) $$\tau_{FRD}=\frac{lim_{a↓60}E\left(y_{i}|age_{i}=a\right)-lim_{a↑60}E\left(y_{i}|age_{i}=a\right)}{lim_{a↓60}E\left(pension_{i}|age_{i}=a\right)-lim_{a↑60}E\left(pension_{i}|age_{i}=a\right)}$$

In Equation (1), $\tau_{\mathrm{FRD}}$ is the average causal effect of treatment, $y_{i}$ is the outcome of interest for individual i, ${\mathrm{age}}_{i}$ denotes the age of individual i, and a represents the cut-off age of 60.

To interpret this result, we also need to define compliance status. Compliers are individuals that would receive treatment if the cut-off were at $age_{i}$ or below, but who would not get the treatment if the cut-off were higher than $age_{i}$. In our study, special interest is given to the effect of the pension income on outcomes for compliers. Compliers in this case are defined as individuals who would receive pension if eligible (aged 60 or older), but not if ineligible (aged below 60). Then we have:

(2) $$\tau_{FRD}=\frac{lim_{a↓60}E\left(y_{i}|age_{i}=a\right)-lim_{a↑60}E\left(y_{i}|age_{i}=a\right)}{lim_{a↓60}E\left(pension_{i}|age_{i}=a\right)-lim_{a↑60}E\left(pension_{i}|age_{i}=a\right)}\;=E\left(y_{i}\left(1\right)-y_{i}\left(0\right)|individual\;i\;is\;a\;complier\;and\;age_{i}=a\right)$$

$y_{i}\left(0\right)\;\mathrm{in}\;\mathrm{Equation}\;\left(2\right)$ denotes the outcomes without exposure to the “treatment” of the NRPS (pension receipt = 0), and $y_{i}\left(1\right)$ denotes the outcomes given the “treatment” of the NRPS (pension receipt = 1).

For each outcome variable $y_{i}$, the RD estimates are calculated using a rectangular kernel with the Calonico et al. (2014) [19] bandwidth selector. Both the reduced-form estimates and the fuzzy RD estimates are reported, along with the bandwidth values. To check the robustness of the reduced-form nonparametric estimation, this study also applies an alternative parametric method using all observations by modeling the estimation as:

(3) $$y_{i}=\alpha +\tau *1\left(age_{i}\geq 60\right)+\beta *f\left(age_{i}-60\right)\;+\delta *1\left(age_{i}\geq 60\right)*f\left(age_{i}-60\right)+\gamma *X_{i}+\epsilon_{i}$$

where $f\left(age_{i}-60\right)$ is a polynomial function of the normalized age. The covariates in $X_{i}$ include gender, educational attainment, household income, and region of residence. To test the sensitivity of the nonparametric fuzzy RD estimates, this study employs the Two-Stage Least Squares (2SLS) method. Equations (4) and (5) illustrate how the 2SLS analysis is carried out in this setting:

First stage:

(4) $$pension_{i}=\alpha_{0}+\pi_{0}*1\left(age_{i}\geq 60\right)+f_{\;}\left(age_{i}-60\right)\;+\gamma_{0}*X_{i}+\varepsilon_{i}$$

Second stage:

(5) $$y_{i}=\alpha_{1}+\tau *pension_{i}+g_{\;}\left(age_{i}-60\right)+\gamma_{1}*X_{i}+\epsilon_{i}$$

Both the reduced-form estimates τ in Equation (3) and the 2SLS estimates τ in Equation (5) are reported in our study.

## 3. Results

### 3.1. First-Stage Estimation for Pension Receipt

**Figure 2:**
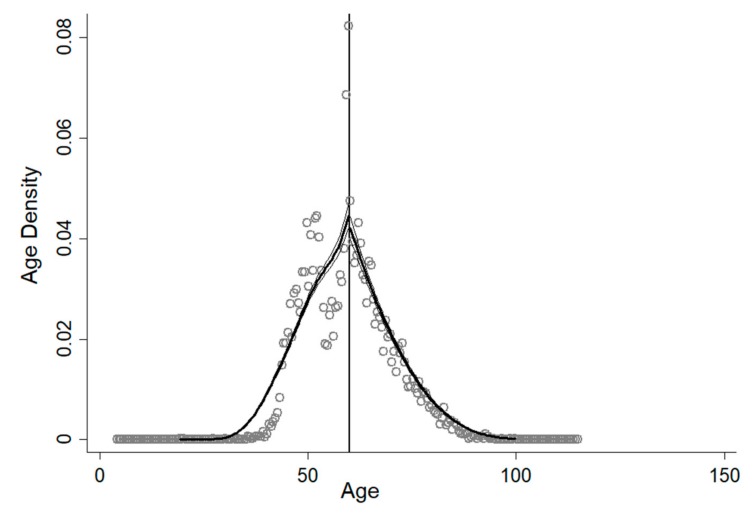
Testing the continuity of age density. Source: China Health and Retirement Longitudinal Survey (2015) [17].

In order for the RD design to be valid, the distribution of age should not be determined in the presence of knowledge about the corresponding cut-point. Otherwise, it can be manipulated to include or exclude specific candidates. To investigate whether the density of age is continuous at the cut-off point of 60, the McCrary (2008) [20] density test is employed. The test result is presented in Figure 2, showing no immediate jump after age 60.

Figure 1 plots the average rate of pension receipt against the cut-off age of 60 using the sample of respondents aged 50 to 70. Figure 1 suggests that aging after the cut-off of 60 sharply increases the likelihood of receiving pension income by approximately 60% for NRPS participants, showing a strong first stage of the fuzzy RD design. To validate the result of this graphical analysis, Table 2 gives the results of both the nonparametric RD regression and the parametric Ordinary Least Square (OLS) estimation with the 2nd-order polynomial of age. Columns (1) and (2) report the nonparametric and parametric RD regression results without covariates, respectively, while Columns (3) and (4) report the regression results when including the covariates. Regardless of whether or not the covariates are included, both nonparametric and parametric RD regressions show a significant jump at the age cut-off of 60. Both of these results suggest that the probability of receiving a pension payment increases (by 56.7% for the nonparametric regression and by 52.9% for the parametric regression) after reaching the pension eligibility age of 60.

### 3.2. Intergenerational Transfers

Pension income could potentially affect intergenerational transfers by increasing elderly financial independence, thus allowing them to be less reliant on monetary support from their adult children. In the CHARLS questionnaire, the elderly were asked to recall the value of all monetary transfers from their adult children (sons and daughters) in the previous 12 months. Table 3 reports the coefficients for the effects of pension income on all the outcomes of interest, including financial support from adult sons (log), financial support from adult daughters (log), co-residence with sons, co-residence with daughters, food consumption expenditure (log), non-food consumption expenditure (log), having no IADL limitation, and self-reported depression index. Both the reduced-form RD estimates and the fuzzy RD estimates using a local linear regression and a 2SLS regression with a 2nd-order polynomial are reported. As shown in Table 3, the first stage F-statistic of the 2SLS regression for the instrumental variable (IV) is statistically significant at 1%, indicating that (age)_i ≥ 60 is a strong IV for pension receipt. In the local linear regression, the optimal bandwidths are selected by applying Calonico et al.’s (2014) [19] method. All the regressions control for gender, educational attainment, household income, and living region.

In Table 3, both the nonparametric and parametric estimations show a significantly negative effect of pension income on money transfer from adult sons. This means that pension income significantly substitutes the monetary support from adult sons. However, regarding the monetary support from adult daughters, only the 2SLS with a 2nd-order polynomial finds a significantly negative effect. Additionally, adult sons respond more to their parents’ increase in income from pension than daughters do. These results indicate that pension income weakens the financial ties between aging parents and their adult children, thus confirming that “altruism” is a primary motivator behind intergenerational transfer. The only children born after the implementation of the one-child policy are beginning to get married and have children; this, coupled with an increase in the average life expectancy, has placed pressure on the traditional Chinese family structure, and the appearance of the “421” family structure has attracted the attention of researchers and policymakers. One of the most important social problems facing the “421” families is the pressure to provide support for four aging parents (parents and parent in-laws). Our estimation results suggest that, financially, the NRPS reduces the burden of elderly support on children, with sons benefiting more than daughters.

As mentioned previously, Cox (1987) [21] shows that private transfers received by lower income individuals are more likely motivated by altruism; in other words, the relatively poor elderly may experience larger reductions in private support than those with higher income as income from other sources increases [22]. To further investigate the effect of pension income on the elderly of different income levels, we divided the whole sample into two subsamples: the poor elderly and non-poor elderly. The poor elderly are those whose income is lower than the rural poverty line. The 2011 rural poverty line of 2300 CNY is applied in this study. Findings presented in Table 4 indicate that pension income only has a significant effect on private transfers for the poor elderly, thus serving as a second confirmation of “altruism” as the motive behind private transfers. Overall, the NRPS is most beneficial for the sons of the poor elderly.

### 3.3. Intergenerational Living Arrangements

The increase in elderly income due to pension receipts could lead to a change in living arrangements, with adult children living separately from their parents in pursuit of education or high-paying jobs in the city. The parametric fuzzy RD estimation results in Table 3 show that the probability of adult sons living with their parents decreases significantly by 6.5% if one parent receives pension. However, we find no similar significant effect for adult daughters. Since adult sons play a more important role in taking care of their aging parents in rural China, pension income would mainly decrease the economic dependence of the elderly on adult sons and enable them to consume more services to replace the care provided by children [6]. Therefore, a significant negative effect is found on sons’ living arrangements. Daughters’ living arrangements are more dependent upon marital status. Due to a patriarchal society, daughters join the husband’s family upon marriage, and are more responsible for taking care of their parents-in-law. As a result, married daughters are less responsive to their own parents’ pension receipts than that of their husbands’ parents [23]. This may explain why we find no evidence that pension income has a significant effect on co-residence decisions of adult daughters.

Economic factors are major forces in determining when adult children leave their parents’ house [24]. The potential effect of pension income on intergenerational living arrangements is also conducted for the poor and non-poor elderly. Similar to the effect on private transfers, pension income has no significant effect on the living arrangements of the non-poor elderly. However, adult sons of the poor elderly are 7.1% less likely to co-reside with their parents once a parent receives pension. Overall, pension income mainly affects the co-residency decisions of adult sons, particularly adult sons of the poor elderly.

### 3.4. Consumption

Most of the elderly population in China, especially in rural China, are financed by savings and/or by transfers from children. With a higher poverty rate, the rural elderly often have to curb their spending on food in order to have enough money for other expenses [25,26]. Although previous findings show that pension income crowds out monetary transfers from adult sons, the NRPS pension payment could directly raise the pensioners’ income. This may then relax their budget constraint, leading to a change in their consumption pattern. Our measure of food expenditure includes both food purchases and self-farmed consumption. In addition to food consumption, we also explored the causal relationship between pension income and non-food expenditure. Table 3 indicates that pension income triggers a 16.3 and 15.1% increase in food and non-food consumption, respectively (parametric fuzzy RD estimation), confirming that pension results in stronger purchasing power. Table 4 shows that the consumption of the non-poor elderly may not be significantly affected by NRPS pension payments. Compared to the generous pension program in South Africa that pays twice the average income [27], the NRPS payment is too low to have a significant effect on wealthier elderly consumption patterns.

### 3.5. Health Outcomes

Pension income could affect the health outcomes of the elderly through multiple transmission channels [7]. One obvious mechanism is that pension income can lead to changes in elderly food consumption, which may further affect their physical health. Diets that are poor in nutrients (e.g., vitamins and phytochemicals) are associated with adverse health outcomes, such as frailty, morbidity, and mortality [28]. Pension income would improve pensioners’ physical health by allowing them to purchase and consume more nutrient-rich food. Another channel is related to living arrangements. While the family support hypothesis posits that elders living alone are at greater risk for depression than those living with a spouse, children, and/or others [29], the family conflict hypothesis argues that co-residence may heighten tensions among family members, thus undermining the advantages of intergenerational support for the elderly [30]. The causal relationship between pension income and intergenerational living arrangements shows that pension income allows for adult children to live apart from their aging parents to pursue better life. As pension income changes traditional forms of living arrangements and intergenerational support for the elderly, we investigate the association between pension income and the psychological well-being of the elderly.

Both physical health measured by the IADL limitation and psychological health measured by the self-reported depression score are estimated by applying a local linear regression and the 2SLS regression with a 2nd-order polynomial. The results in Table 3 show no significant effect on physical health, but the coefficients in different regressions are negative, indicating that average physical health is worse among those who are older. This is consistent with how physical health deteriorates with age. Our research shows that pension income increases elderly food consumption, which is predicted to improve physical health. However, many studies show that the elderly in rural China still need to work long hours and do so until they are no longer physically capable, adversely affecting physical health [7,31]. The insignificant negative effect of pension income on physical health reveals that the improvement of physical health due to the rise in food consumption does not offset the adverse effects of physically demanding work and age. Regarding psychological health, we only find a significantly negative effect in the parametric fuzzy RD estimation, and pension income decreases the self-reported depression score by 1.04, approximately 11.3% of the mean value. Although pension income decreases the likelihood that adult sons co-reside with their aging parents, which may have a negative effect on psychological health according to the family support hypothesis, pension income also enables the elderly to purchase more formal care services to replace the care provided by adult children. The overall association between pension income and psychological health is positive. When dividing the whole sample into the poor and non-poor elderly, we still only find significant effects on the poor elderly.

Generally, the NRPS benefits adult children by reducing their financial burden of supporting their aging parents and providing them with greater flexibility to participate in the job market [5]. Our results indicate that sons, who carry more responsibility for taking care of their elderly parents, are more responsive to their parents’ pension income than daughters are. The NRPS substitutes part of the financial support from adult sons and also reduces the probability that adult sons live with their parents. However, we do not find any significant effect of pension income on the likelihood that adult daughters co-reside with their parents. Additionally, the results of measuring well-being for aging parents show that both consumption and health are improved due to pension payments. When considering income level, the NRPS only significantly affects the poor elderly, which is not surprising considering that a modest pension income matters more for the elderly with lower income [7].

### 3.6. Robustness Checks

For more confidence in the estimation results, a series of robust checks were incorporated. With a given window of bandwidth around the cut-point, a fuzzy RD model can be estimated straightforwardly—however, choosing this bandwidth is challenging. The tradeoff between bias and precision is a major motivator of bandwidth selection—larger bandwidths reduce the noisiness of estimates at the expense of introducing bias from data points far from the cut-off for treatment. To test the robustness of our RD estimation results, besides a sample of elderly aged within 10 years of the 60 cut-off for the OLS with a 2nd-order polynomial, we also duplicated the estimation procedures, applying samples of elderly aged 55–65 and 45–75. The estimates using different bandwidth specifications are consistent, confirming that our results are robust to bandwidth selection (Table A1). To test whether the discontinuities are driven by the receipt of pension income, rather than other age-related changes, the RD model was repeated using different cut-offs. Imbens and Lemieux (2008) [18] suggested a way of implementing a test for jumps at the median of the two subsamples on either side of the cut-off value, and age 54 and 64 were used as two alternative cut-offs. On applying different cut-offs using a 2nd-order polynomial, none of the RD estimates were significant for outcome variables (Table A2). This confirms that the discrete change at age 60 is driven by the pension income.

## 4. Discussions

### 4.1. Pension Income and Intergenerational Relationships

Due to deep-rooted Confucian “Filial Piety”, informal family support, which is characterized by both pecuniary and non-pecuniary (psychological) support provided by adult children to their parents, has been China’s age-old security system for a long time [32]. The implementation of the NRPS, sponsored by the Chinese government, has sparked a departure from the traditional family support system to a new period of social pension for rural China. Payments through the NRPS directly raise pensioners’ income, potentially spurring a change in intergenerational relationships. Economic theory predicts that intergenerational transfers decrease with recipients’ income if they are motivated by altruism [33,34], but increase with income if they are motivated by an exchange [35]. Altruistic intergenerational transfers occur when adult children care about their parents’ utility, whereas transfers motivated by an exchange are regarded as a form of repayment for previous investments by parents, or as a way to compensate parents for providing care to adult children [36]. Cox (1987) [21] found that private transfers are more likely motivated by altruism if the recipient’s income is low, while exchange as a motivation dominates at higher income levels [22]. In poor rural areas, we found that intergenerational transfers are more motivated by altruism, and additional income from the NRPS decreased the financial support provided by adult children to elderly parents. The NRPS reduced the financial burden of adult children, and therefore parents’ participation in NRPS also benefit their children.

In addition to financial support, living arrangements have long been regarded as the foundation of elderly care [37]. Traditionally in rural China, the elderly live with family members so that they can easily receive care from their adult children or extended family [38]. Pension income from the NRPS increases elderly parents’ economic independence and enables them to live separately with adult children [6]. If considering living arrangement as a form of consumption, additional economic resources provided by pension income could also explain a decreased consumption of household public goods, and the decision to live apart. With limited economic resources, living with family members to share household public goods could enjoy the economies of scale. However, with the relaxation of household financial constraint, sharing public goods becomes less economical and family members choose to live separately. Living independently encourages adult children to migrate to urban areas to pursue better lives. For elderly parents, living independently may avoid potential intergenerational conflict between family members and provide a better quality of life for elderly parents [38,39]. However, a burgeoning literature documents an association between living arrangements and the psychological well-being of the older adults [40,41]. As an important factor to affect well-being, living arrangements have long been regarded as a predictor of social network patterns and social support to the elders. It is generally believed that coresidential arrangements are better than solitary arrangements in protecting the psychological health of older persons. In our analysis, the increasing purchasing power as a result of pension income reduced the likelihood that adult sons live with their parents. This change of living arrangement could affect the well-being of both elderly parents and adult sons.

### 4.2. Pension Income, Consumption, and Health

The consumption expenditures of the elderly in China, especially in rural China, are largely financed by transfers from adult children and/or personal savings. However, as a result of the birth control policy in the last three decades, the elderly dependency ratio has dramatically increased at a relatively low income level. As China continues to age, the elderly are increasingly experiencing poverty and deprivation of basic services. Social pension programs have been widely regarded and implemented as an important policy tool for providing the elderly with income security. Their success in stimulating consumption has also been confirmed by many studies [6,42]. The NRPS “windfall income” relaxes the budget constraints of the elderly, leading to the change in spending behavior. Shu (2018) [43] showed that although the NRPS pension benefits could not suffice in allowing elderly people to maintain a normal quality of life, for the lowest income group, they do account for a large proportion of the total income. Our finding is consistent with previous studies, showing that the NRPS pension income represents a larger relative relaxation of the budget constraint for the elderly with lower income, and affects the consumption of the elderly with lower income more than those with higher income.

The NRPS pension payments influence elderly health in various ways. Pension income allows for the consumption of more protein-rich food, a lack of which is found to be strongly associated with adverse health outcomes such as frailty, morbidity, cognitive impairment, depression, and mortality [7]. Additionally, the elderly are less likely to participate in physically demanding work that adversely affects health when provided with pension income [44]. Furthermore, a change in elderly living arrangements could also be an important determinant of their health. Although much research has been conducted on this relationship, conclusions remain ambiguous. From the family support perspective, co-residence can protect elderly parents from social isolation and depression [45]. However, family conflict hypothesis posits co-residence could also heighten tension among family members [30]. Therefore, investigating how the NRPS pension payments affect health would benefit policymakers by providing effective and specific policy advice for improving elderly well-being. Results in Table 3 indicate that pension income significantly improves the psychological health status of the aging parents.

## 5. Conclusions

As aging creates great challenges for traditional family-based eldercare in rural China, social pension programs are being considered by the government to ensure the well-being of the growing elderly population. The NRPS, which was launched in 2009, is a pension program targeting the rural elderly population. Employing both the reduced-form RD estimates and the fuzzy RD estimates using a local linear regression and a 2SLS regression with a 2nd-order polynomial, this paper investigates the impact of pension income on the well-being of the elderly and their adult children. The well-being of the elderly is measured by consumption and health, while the well-being of adult children is measured by private transfers and intergenerational living arrangements. Our results indicate that sons, who are more responsible for taking care of their aging parents, are more responsive to their parents’ pension income than daughters. Pension income crowds out approximately 27.9% of the monetary support from adult sons and decreases the likelihood of co-residency between adult sons and old parents by 6.5%. Regarding the well-being of parents, the results show that pension income promotes food and non-food consumption by 16.3 and 15.1%, respectively, due to the stronger purchasing power that arises as a result of pension income. The association between pension income and psychological health is significantly positive, but no significant effect is found on physical health. To capture the difference and relative importance of pension payments for the elderly of different income levels, we divided the whole sample into two subsamples: the poor elderly and the non-poor elderly. Our results show that pension payments only have a significant effect on the poor elderly, which may be explained by the fact that the current payment amount is still too small to have a significant effect on the wealthier elderly.

Our empirical findings hold important policy implications. First, the findings suggest that pension payments to elderly parents raise their income, which benefits both adult children, especially adult sons, and elderly parents. Our findings also confirm the important role of social pension programs in dealing with the challenges associated with an aging population. Therefore, establishing and improving the social support system for the elderly should be regarded as a significant way to mitigate the adverse effects of population aging on economic and social development. Secondly, due to China’s large population and less developed economy, the NRPS pension payment can only provide basic support for the elderly, with the poor elderly benefitting more from this pension program than the non-poor elderly. In light of the differences in the impact of pension income on high- and low-income groups, we suggest further improving the fiscal subsidy mechanism and increasing the pension subsidy for the poor elderly. The non-poor elderly should also be encouraged to purchase commercial pension insurance and increase their awareness of self-security. Although the results indicate that social pension income decreases the likelihood that adult children and their parents live together, which may adversely affect psychological health, a positive relationship between pension income and psychological health was found. This may be explained by the fact that pension income provides parents with more economic resources, which enables them to consume more services to replace the care provided by children [34]. Family-based elderly care is the main support for the rural elderly, and community- and institution-based services are still very weak. With more and more adult children living apart from their parents, demand for community services and institutional care increases. Thus, it is important to improve pension service institutions and accelerate the integration of pension, medical, education, rehabilitation and other public service resources.

In this study, we only focus on the impacts of social pension on support provided from adult children to elderly parents. It should also be noted, however, that an increase in pension income causes a decline in the old-age labor supply [2,10]. Therefore, old parents could spend more time on taking care of grandchildren and possibly provide financial support for their children or grandchildren. To capture a broader picture of the impact of pension income, future studies should consider support from elderly parents to adult children. Inspired by current studies, future research could also investigate the implications of pension programs on the career choices and migration of the younger generation.

## Figures and Tables

**Table 1:** Variable definitions and summary statistics.

Variable	Definition	Mean	SD
NRPS	If the respondent received pension payment (=1)	0.461	0.499
Male	If the respondent is male (=1)	0.470	0.499
Age	Age of the respondent	60.05	10.31
Illiterate	If the respondent has no formal education (=1)	0.280	0.449
Elementary	If the respondent has primary education (=1)	0.393	0.489
Junior	If the respondent has junior high school education (=1)	0.161	0.367
Senior	If the respondent has senior high school education (=1)	0.048	0.214
Income	Household income (1000 CNY)	12.94	106.3
Food	Daily food consumption expenditure (yuan/day)	14.95	53.8
Non-Food	Daily non-food consumption expenditure (yuan/day)	17.45	68.26
IADL	If no IADL limitation (=1)	0.741	0.438
Depression	The sum of the mental well-being measurement score	9.212	5.709
Transfer_son	Transfer from adult son	1261	4638
Transfer_daughter	Transfer from adult daughter	681.5	3162
Coresident_son	If the respondent co-resides with son (=1)	0.323	0.468
Coresident_daughter	If the respondent co-resides with daughter (=1)	0.065	0.247
*N*	No. of observations	9324	

Source: China Health and Retirement Longitudinal Survey (2015) [17]. Note: NRPS: New Rural Pension Scheme; IADL: instrumental activities of daily living; SD: Standard Deviation.

**Table 2:** First-stage estimates for pension receipt.

Depend Variable	Local Linear Regression	OLS with a 2nd-Order Polynomial	Local Linear Regression	OLS with a 2nd-Order Polynomial
	(1)	(2)	(3)	(4)
Indicator of pension receipt (Yes = 1; No = 0)	0.529 *** (0.025)	0.511 *** (0.020)	0.567 *** (0.023)	0.554 *** (0.018)
Bandwidth	5.53		6.45	
Covariate	No	No	Yes	Yes
*N*	7705			

Source: China Health and Retirement Longitudinal Survey (2015) [17]. Note: Sample consists of respondents aged within 10 years of the cut-off (60). Columns (1) and (2) reports the local linear regression and the OLS estimation with the 2nd-order polynomial of the running variable, respectively. Columns (3) and (4) shows the estimation results when including covariates. Covariates include gender, educational attainment, household income, and region of residence. Robust standard errors are in parentheses. *** *p* < 0.01.

**Table 3:** Regression discontinuity (RD) estimation on outcomes of Interest.

Dependent Variable	Local Linear Regression		2SLS with a 2nd-Order Polynomial	
	Reduced-Form RD	Fuzzy RD	Reduced-Form RD	Fuzzy RD
Transfer_son	−0.146 *	−0.291 *	−0.204 **	−0.279 **
Bandwidth	9.412	7.573		
Transfer_daughter	−0.098	−0.152	−0.148 *	−0.203 **
Bandwidth	8.899	8.047		
Coresident_son	−0.101 ***	−0.135 ***	−0.047 **	−0.065 **
Bandwidth	5.826	7.466		
Coresident_daughter	0.018	0.033	0.021	0.029
Bandwidth	6.318	5.613		
Food	0.112 *	0.170 *	0.118 **	0.163 **
Bandwidth	7.769	8.585		
Non-food	0.098 *	0.148 *	0.109 **	0.151 **
Bandwidth	7.800	8.233		
IADL	−0.018	−0.025	−0.020	−0.027
Bandwidth	6.470	7.101		
Depression	−0.437	−0.719	−0.758 **	−1.037 ***
Bandwidth	7.397	7.132		
First stage F-statistics for IV				3742.46
*N*	7705			

Source: China Health and Retirement Longitudinal Survey (2015) [17]. Note: Table 3 presents the reduced-form RD estimates and the fuzzy RD estimates using local linear regression and a Two-Stage Least Squares (2SLS) method with a 2nd-order polynomial, respectively. In the local linear regression, the optimal bandwidths are selected by applying Calonico et al.’s (2014) [19] method. All the regressions control for gender, educational attainment, household income, and living region. * *p* < 0.1, ** *p* < 0.05, and *** *p* < 0.01.

**Table 4:** RD estimation on outcomes of interest by poverty status.

Dependent Variable	Poor Elderly				Non-Poor Elderly			
	Local Linear Regression		2SLS with a 2nd-Order Polynomial		Local Linear Regression		2SLS with a 2nd-Order Polynomial	
	Reduced-Form RD	Fuzzy RD	Reduced-Form RD	Fuzzy RD	Reduced-Form RD	Fuzzy RD	Reduced-Form RD	Fuzzy RD
Transfer_son	−0.238 *	−0.426 *	−0.213 **	−0.281 **	0.041	0.025	−0.182	−0.278
Bandwidth	7.212	6.558			6.574	7.615		
Transfer_daughter	−0.105	−0.167	−0.197 **	−0.260	−0.046	−0.082	−0.005	−0.008
Bandwidth	7.877	7.078			6.886	8.864		
Coresident_son	−0.124 ***	−0.190 ***	−0.054 **	−0.071 **	0.015	0.021	−0.008	−0.013
Bandwidth	6.661	7.262			7.524	8.237		
Coresident_daughter	0.029	0.059	0.026	0.035	−0.012	−0.011	0.001	0.001
Bandwidth	5.566	4.970			6.265	7.394		
Food	0.138 *	0.254 *	0.165 *	0.221 *	0.087	0.150	−0.036	−0.054
Bandwidth	6.258	5.949			6.275	6.799		
Non-food	0.173 ***	0.308 ***	0.153 **	0.204 **	−0.143	−0.041	−0.052	−0.081
Bandwidth	8.034	7.337			6.416	8.061		
IADL	0.004	0.003	0.026	0.034	−0.063	−0.084	−0.006	−0.009
Bandwidth	7.615	6.795			6.552	7.636		
Depression	−0.245	−0.493	−0.732 **	−0.966 **	−0.726	−1.106	−0.657	−1.008
Bandwidth	6.381	6.673			6.440	7.590		
First stage F-statistics for the instrumental variable (IV)				2974.42				783.89
*N*	5292				2413			

Source: China Health and Retirement Longitudinal Survey (2015) [17]. Note: * *p* < 0.1, ** *p* < 0.05, and *** *p* < 0.01.

## Appendix A

**Table A1:** Robustness check for alternative bandwidths.

Dependent Variable	Aged between 55 and 65				Aged between 45 and 75			
	Poor Elderly		Non-Poor Elderly		Poor Elderly		Non-Poor Elderly	
	Reduced Form RD	Fuzzy RD	Reduced Form RD	Fuzzy RD	Reduced Form RD	Fuzzy RD	Reduced Form RD	Fuzzy RD
Transfer_son	−0.246 *	−0.316 *	0.133	0.193	−0.184 *	−0.242 *	−0.048	−0.078
Transfer_daughter	0.008	0.010	−0.041	−0.059	−0.019	−0.025	−0.017	−0.027
Coresident_son	−0.223 ***	−0.287 ***	−0.010	−0.014	−0.057 *	0.075 *	−0.023	−0.039
Coresident_daughter	0.035	0.049	0.022	0.035	0.020	0.026	−0.001	−0.001
Food	0.239 *	0.309 *	0.024	0.157	0.170 **	0.228 **	0.025	0.040
Non-food	0.286 ***	0.372 ***	0.094	0.132	0.175 ***	0.234 ***	−0.101	−0.083
IADL	0.038	0.049	0.085	0.123	0.004	0.005	−0.050	−0.081
Depression	−0.331*	−0.426 *	−1.600	−1.157	−0.503 *	−0.663 *	−0.827	−1.340
First stage F-statistics for IV	127.43	44.50	2346.93	470.07
*N*	1952		772		6287		2958	

Source: China Health and Retirement Longitudinal Survey (2015) [17]. Note: Samples aged 55–65 and 45–75 are applied in the OLS regression with a 2nd-order polynomial, respectively. * *p* < 0.1, ** *p* < 0.05, and *** *p* < 0.01.

**Table A2:** Robustness check for alternative cut-offs.

Dependent Variable	Cut-off Age of 54				Cut-off Age of 64			
	Poor Elderly		Non-Poor Elderly		Poor Elderly		Non-Poor Elderly	
	Reduced Form RD	Fuzzy RD	Reduced Form RD	Fuzzy RD	Reduced Form RD	Fuzzy RD	Reduced Form RD	Fuzzy RD
Transfer_son	0.267	0.093	0.097	0.029	−0.141	−0.120	0.045	0.057
Transfer_daughter	0.145	0.050	0.046	−0.014	−0.025	−0.021	−0.014	−0.015
Coresident_son	0.043	0.015	−0.066	0.020	0.026	0.022	0.012	0.015
Coresident_daughter	0.015	0.052	0.090	0.027	−0.027	−0.023	−0.082	−0.104
Food	0.102	0.196	0.089	−0.267	0.035	0.269	0.026	−0.093
Non-food	0.086	0.109	0.038	−0.065	0.008	0.064	0.017	0.016
IADL	0.045	0.041	−0.021	0.062	−0.035	−0.030	−0.046	−0.058
Depression	−0.341	−1.192	−0.221	−0.666	1.024	−0.867	−0.915	−1.161
First stage F-statistics for IV	0.126	1.513	1.729	0.275
*N*	2968		1314		2324		1099	

Source: China Health and Retirement Longitudinal Survey (2015) [17]. Note: Age 54 and 64 were used as two alternative cut-offs for the OLS regression with a 2nd-order polynomial.
